# Multifaceted Role of Copper Homeostasis in Gut Health: From Molecular Mechanisms to Therapeutic Interventions

**DOI:** 10.3390/cells15060545

**Published:** 2026-03-19

**Authors:** Fucheng Lu, Xuchen Wang, Xiaoyan Xue, Liqiang Liu, Dongmei Li, Anfang Liu, Simeng Qin, Lingbin Liu

**Affiliations:** College of Animal Science and Technology, Southwest University, Beibei, Chongqing 400715, China; 18178001293@163.com (F.L.); m13920074889@163.com (X.W.); luvsic177@163.com (X.X.); liuliqiang0207@163.com (L.L.); ldm0175@163.com (D.L.); liuanfang@swu.edu.cn (A.L.)

**Keywords:** copper, copper homeostasis, gut health, intestinal immunity, dysregulation of copper homeostasis

## Abstract

**Highlights:**

**What are the main findings?**

- This review delineates the molecular machinery of intestinal copper absorption and transport, focusing on key transporters and regulatory mechanisms.
- We elucidate how copper homeostasis acts as a central modulator of gut iron metabolism, barrier integrity, mucosal immunity, the microbiome, and the gut–brain/liver axes.

**What are the implications of the main findings?**

- The pathophysiological roles of copper dyshomeostasis in Menkes disease, Wilson disease, and intestinal cancers are comprehensively discussed.
- We explore the therapeutic potential and mechanisms of copper-targeting agents in oncology, evaluating current strategies and future directions.

**Abstract:**

Intestinal copper homeostasis governs gut health through its dual roles as an enzymatic cofactor and signaling mediator. This review discusses the molecular basis of copper absorption/transport, genetic regulation, and its functional impacts. Copper-dependent enzymes maintain intestinal barrier function and metabolism, while copper availability shapes the composition of gut microbiota and mucosal immunity. The dysregulation of copper homeostasis, specifically pathological accumulation, contributes to the development of CRC by inducing dysbiosis of gut microbiota, chronic inflammation, and metastasis. This review systematically evaluates copper-targeted therapies and the associated unresolved challenges. Future efforts should prioritize defining cell-specific copper handling, metal interaction networks, and the copper–gut microbiota–immune axis in non-cancer pathologies. Moreover, future studies should also focus on developing stratified biomarker panels and spatially precise interventions to harness copper biology for diagnostic and therapeutic innovation.

## 1. Introduction

Copper is an essential trace element for all eukaryotes, serving as an indispensable cofactor for enzymes involved in aerobic respiration and antioxidant defense [1]. However, the redox activity of copper presents a dual threat: excessive Cu^+^/Cu^2+^ ions catalyze the generation of Reactive Oxygen Species (ROS), causing damage to DNA, lipids, and proteins [2], while their direct binding to lipid-acylated Tricarboxylic Acid (TCA) cycle proteins induces aggregation, loss of iron–sulfur clusters, and cuproptosis [3]. These dual effects necessitate evolutionarily conserved homeostatic mechanisms to balance the vital functions against their inherent toxicity.

In daily diets, the main dietary sources of copper are generally nuts, whole grains, seafood, and organ meats [4], with an approximate daily intake of 0.9 mg for adults [5]. Copper metabolism in the human body is a systemic process involving the coordinated action of multiple organs. The intestine serves as the primary site of copper absorption and initial regulatory hub, controlling the uptake and transport of dietary copper into the portal circulation [6]. The liver functions as the central hub of copper metabolism, receiving copper via the portal vein, where it is stored, incorporated into copper-dependent proteins, and systemically distributed through biliary excretion and bloodstream regulation [7]. The kidneys contribute to copper homeostasis through renal excretion and reabsorption mechanisms [8]. Additionally, copper is utilized and stored in various organs, including the brain, heart, bones, and skeletal muscles, where it plays essential roles in physiological functions [6]. Copper homeostasis imbalance can lead to either deficiency or excess, resulting in multisystem clinical manifestations. Copper deficiency may be classified as hereditary (e.g., Menkes disease) or acquired, with the latter being more common. It is frequently misdiagnosed as myelodysplastic syndrome or vitamin B12 deficiency, and delayed treatment may lead to irreversible neurological damage. Copper excess is primarily associated with the genetic disorder Wilson disease.

The triple function of the intestine, including absorption, excretion, and storage, establishes it as the “first gateway” for systemic copper homeostasis [9,10]. Critically, the local intestinal copper contents further modulate host health by shaping the composition of gut microbiota, as well as immune microenvironments: the physiological levels sustain symbiosis, while excess copper activates bacterial efflux pumps, thereby fostering antibiotic resistance and dysbiosis [3,11].

Copper homeostasis is dynamically regulated by transporters, chaperones, and chelators and maintains intestinal integrity [12]. Its disruption shows multifaceted pathology. Dysregulated copper can impair iron absorption via iron–sulfur cluster inhibition, inducing anemia [10], and microbial dysbiosis promotes inflammation [13]. Consequently, excessive copper accumulation can initiate a vicious cycle.

Gastrointestinal disorders are heterogeneous and fall into three main categories: inflammatory, infectious, and metabolic, with distinct etiologies and clinical traits. Inflammatory Bowel Disease, a chronic gastrointestinal inflammatory condition comprising Crohn’s disease and ulcerative colitis, arises from complex interactions between genetic susceptibility, environmental triggers, gut dysbiosis, and dysregulated immunity. This triggers chronic inflammation, barrier damage, and tissue injury, marked by relapsing-remitting courses and intestinal/extra-intestinal manifestations linked to microbial and metabolic shifts [14,15]. Infectious enteritis is acute intestinal inflammation caused by pathogenic bacteria, viruses, parasites or toxins, presenting with diarrhea, abdominal cramps and fever, transmitted via contaminated sources or person-to-person contact; it is generally acute but severe in immunocompromised patients. Metabolic gut disorders, encompassing inherited and acquired types, involve gut-metabolism crosstalk: inherited forms include lactose intolerance, while acquired ones feature non-alcoholic fatty liver disease and diabetes-related gut dysfunction. Notably, disrupted host-microbial metabolism is also a key IBD pathophysiological feature.

In inflammatory states, it induces ROS overproduction and intestinal barrier dysfunction, while in CRC, elevated copper levels drive tumor progression via pro-angiogenic signaling and cuproptosis [2,16]. Cuproptosis, a newly identified form of copper-dependent cell death, plays a complex and critical role in the initiation, progression, and treatment of intestinal cancers, particularly CRC. The core mechanism involves the excessive accumulation of copper ions (Cu^2+^) within cells, which subsequently targets lipoylated tricarboxylic acid (TCA) cycle proteins in the mitochondrial respiratory chain (e.g., DLAT). This leads to the aberrant aggregation of these proteins, disruption of iron–sulfur cluster protein stability, and eventual mitochondrial dysfunction, culminating in proteotoxic stress and cell death [17,18]. Therefore, understanding the complex interactive mechanisms among copper homeostasis, gut physiology, and intestinal disease pathogenesis is crucial for developing novel therapeutic strategies.

## 2. Molecular Mechanisms Governing Intestinal Copper Homeostasis

### 2.1. Luminal Reduction and Apical Uptake

**Figure 1:**
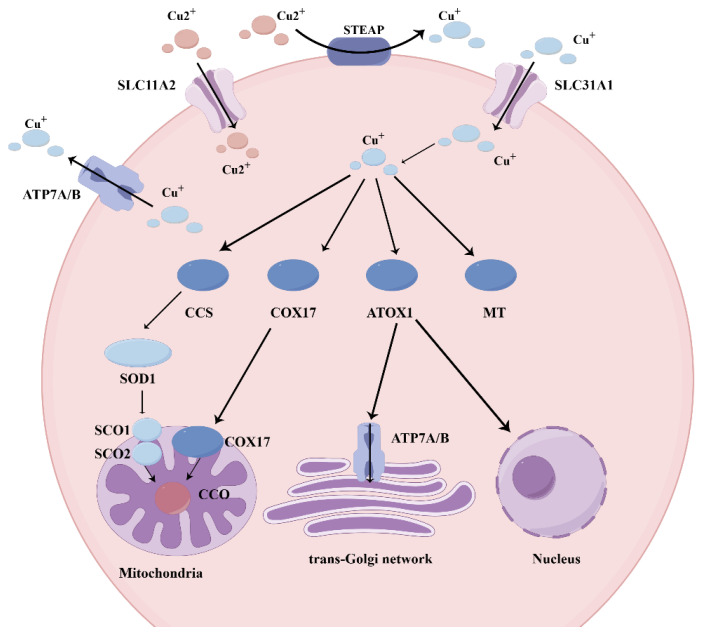
A diagram of cellular copper transport and metabolism. Abbreviations: ATP7A: ATPase Copper Transporting Alpha; ATP7B: ATPase Copper Transporting Beta; SLC31A1: Solute Carrier Family 31 Member 1; SLC31A2: Solute Carrier Family 31 Member 2; STEAP: Six-Transmembrane Epithelial Antigen of the Prostate; CCS: Copper Chaperone for Superoxide Dismutase; COX17: Cytochrome c Oxidase Copper Chaperone 17; ATOX1: Antioxidant 1 Copper Chaperone; MT: Metallothionein; SOD1: Superoxide Dismutase 1; SCO1: Synthesis of Cytochrome C Oxidase 1; SCO2: Synthesis of Cytochrome c Oxidase 2; CCO: Cytochrome c Oxidase.

The human body acquires copper primarily from dietary sources and drinking water. It is predominantly absorbed in the duodenum, with additional uptake along the small intestine [19]. Its absorption requires the enzymatic reduction of Cu^2+^ to Cu^+^, a process mediated by specific metallo-reductases, including the Six-Transmembrane Epithelial Antigen of the Prostate (STEAP) family [20,21]. Cu^+^ is primarily transported across cellular membranes in intestinal and somatic tissues via two major copper permease transporters, including Solute Carrier Family 31 Member 1/Copper Transport protein 1 (SLC31A1/CTR1) and SLC31A2/CTR2 of the SLC31 copper transporter family [22]. CTR1 functions as a high-affinity Cu^+^ importer and has highly conserved structural and functional features among eukaryotes. It is localized to the plasma membrane and intracellular vesicles, highlighting its importance in maintaining cellular copper homeostasis [23]. It is localized to the apical membrane of enterocytes in diverse species, including *Drosophila melanogaster*, mice, rats, and pigs [23,24,25,26]. Further studies have shown that CTR1 is distributed not only on both apical and basolateral membranes but also within intracellular compartments of enterocytes [26]. Moreover, its expression is dynamically regulated by copper availability: it degrades under high copper concentrations to prevent toxicity, while the increased membrane trafficking promotes copper uptake during deficiency [27]. SLC11A2/Divalent Metal Transporter 1 (SLC11A2/DMT1)-mediated compensatory uptake may bypass the functional impairment of SLC31A1 (Figure 1) [28].

### 2.2. Intracellular Buffering and the Labile Copper Pool

Free copper displays potent cytotoxicity; therefore, following cellular uptake, copper ions are either incorporated into copper-dependent enzymes or stored in non-toxic forms. The intracellular free copper concentrations are strictly maintained at minimal levels through high-affinity chelation by Metallothioneins (MTs) and Glutathione (GSH) [29]. GSH is a tripeptide comprising glutamate, cysteine, and glycine residues. GSH plays two essential roles in copper homeostasis: (1) it serves as the major intracellular copper buffer via direct ion chelation and (2) it functions as the likely initial copper acceptor following cellular uptake. The resulting copper–GSH complex can subsequently deliver Cu^+^ ions either to specific chaperones for biological incorporation or to MTs for detoxification [30,31,32].

Within cells, oxidation-state-specific labile copper pools exist in both Cu^+^ and Cu^2+^ forms, functioning as dynamic signaling molecules that participate in the regulation of diverse biological processes. Copper exhibits heterogeneous distribution among organelles, establishing subcellular concentration gradients. Lysosomes serve as major copper storage compartments, with approximately 57.3% of cellular copper localized in lysosome-enriched large granule fractions in bovine hepatocytes [33]. Lysosomal copper mobilization depends on the coordinated action of the autophagy–lysosome pathway and the copper transporter Atp7a. In senescent cells, reduced Atp7a expression and impaired autophagic function lead to lysosomal copper accumulation. Activation of autophagy by rapamycin restores copper efflux capacity in aging cells, where copper accumulation is accompanied by diminished lysosomal degradative function. The synergistic regulation of copper homeostasis by Atp7a and the autophagy–lysosome pathway is further evidenced by exacerbated copper accumulation in Atp7a-deficient senescent cells such as Mobr MEFs [34]. Mitochondria harbor a chemically reactive Cu^2+^ pool that sustains metabolic and epigenetic reprogramming by catalyzing NAD(H) redox cycling, thereby promoting inflammatory responses [35]. This organelle-specific copper pool operates independently from lysosomal storage and contributes to distinct physiological functions.

### 2.3. Copper Chaperone Networks and Mitochondrial Delivery

The intracellular copper trafficking is precisely regulated through specialized chaperone proteins, including the Copper Chaperone for SOD (CCS). This metallochaperone specifically delivers copper ions to their cognate targets, such as the cytoplasmic Cu/Zn-SOD1 [36,37]. CCS catalyzes the dismutation of superoxide radicals into oxygen and hydrogen peroxide. The antioxidant enzyme SOD1 is localized primarily in the cytoplasm, as well as in a minor fraction in mitochondrial intermembrane space.

The Cytochrome c Oxidase Copper Chaperone 17 (COX17), a copper chaperone, can specifically deliver copper ions to the mitochondrial membrane proteins, including Synthesis of Cytochrome C Oxidase 1 (SCO1) and SCO2, which are essential for the COX assembly [38]. The COX assembly contains two catalytic subunits: COX1 and COX2. These subunits interact with copper at distinct active sites: COX1 at the Cu_B_ center and COX2 at the Cu_A_ site [39]. The mitochondrial copper chaperone COX17, which is localized to the intermembrane space, can mediate the transfer of cytoplasmic copper to the inner membrane for COX biogenesis. [40,41]. Additionally, COX17 also delivers copper to the COX1 subunit via COX11 [38,42].

### 2.4. Basolateral Export and Systemic Distribution

The P-type ATPases, including ATP7A and ATP7B, function as the major cellular copper efflux transporters. These ATPases share about 60% sequence homology and coordinately regulate systemic copper homeostasis [43]. The cytoplasmic Cu^+^ delivery to both transporters at the Trans-Golgi Network (TGN) is mediated by copper chaperone Antioxidant 1 Copper Chaperone (ATOX1) [44]. ATP7A undergoes continuous recycling between the TGN and plasma membrane, while ATP7B transfers between the TGN and cytoplasmic vesicles [45,46]. ATP7A is constitutively localized at the TGN in most tissues and supplies copper for cuproenzyme biosynthesis [46,47], relocating to the plasma membrane during copper excess to mediate efflux [44,48].

In the intestinal lumen, reduced copper enters enterocytes primarily via CTR1, binds to ATOX1, and is exported into the circulation by ATP7A. In contrast, the liver, acting as a central metabolic hub, also takes up copper through CTR1 but distributes it to key enzymes such as superoxide dismutase via chaperones including CCS and SCO1. The key distinction lies in the handling of copper excess: hepatocytes store copper through metallothioneins or excrete it into bile via ATP7B, whose vesicular trafficking is dynamically regulated by intracellular copper levels. Intestinal epithelial cells lack this copper-responsive regulatory mechanism, underscoring a fundamental difference in copper homeostasis between absorptive and regulatory tissues [49].

### 2.5. Transcriptional and Post-Translational Regulation

The expression of copper transporters is dynamically modulated by copper availability. Current studies indicate two distinct mechanisms modulating *SLC31A1* expression and function in copper absorption: (1) post-translational regulation through copper-dependent endocytosis and degradation [50] and (2) the SLC31A1 regulatory network is a multidimensional dynamic system driven by Sp1 as the core transcriptional regulator, mediated by HIFs for hypoxic adaptation, modulated by MTF-1 in response to metal stress, finely tuned by microRNAs at the post-transcriptional level, and integrated with inflammatory factors that transduce microenvironmental signals [51,52,53].

The apical-basal polarity of intestinal epithelial cells underpins their barrier and absorptive functions, with the polarized localization and trafficking dynamics of copper transporters such as ATP7A and ATP7B playing critical roles. Under low copper conditions, both ATP7A and ATP7B reside in distinct subdomains of the trans-Golgi network (TGN). Upon copper stimulation, ATP7A traffics to the basolateral membrane, whereas ATP7B translocates to the apical membrane via recycling endosomes, apical sorting endosomes, and apical recycling endosomes [54]. The clathrin adaptor protein complex 1 (AP-1) serves as a core regulator of this polarity. Knockout of AP-1 disrupts ATP7A polarity, resulting in its mislocalization to both apical and basolateral membranes, while ATP7B, although losing TGN retention, retains apical membrane localization in a copper-independent manner [54]. Auxiliary mechanisms support polarity maintenance. Vesicle tethering complexes, such as the exocyst complex, contribute to apical-basal polarity by anchoring secretory vesicles to specific membrane domains [55]. V0-ATPase maintains brush border integrity and apical polarity modules by regulating RAB-11-positive endosomes and SNAP-29-mediated trafficking; its disruption leads to microvillus inclusion disease-like phenotypes [56]. Transcytosis, exemplified by basolateral-to-apical transport of CFTR, serves as a corrective mechanism for polarity errors, rescuing mis-sorted proteins from basolateral mistargeting [57].

Microenvironmental gradients along the crypt-villus axis, such as bone morphogenetic protein (BMP) signaling, regulate enterocyte differentiation and influence copper transporter expression and function. Spatially, stem cells and progenitor cells at the crypt base predominantly express the copper importer CTR1, while differentiated enterocytes at the villus tip exhibit high expression of copper exporters ATP7A and the C. elegans homolog CUA-1 [58]. BMP signaling, which increases along the villus axis, drives functional zonation by regulating transcriptional programs: crypt cells prioritize proliferation, whereas villus cells specialize in absorption and metal efflux [59]. Dynamic regulation maintains copper homeostasis. Under copper deficiency, CUA-1 localizes to the basolateral membrane to facilitate efflux into the circulation; under copper overload, it relocates to lysosome-related organelles such as gut granules for sequestration and detoxification. An isoform, CUA-1.2, lacking the N-terminal domain, is constitutively targeted to the basolateral membrane, providing basal efflux capacity [58]. Disruption of the crypt-villus gradient, such as through defective BMP signaling, leads to dysregulated copper transporter expression and impaired systemic copper homeostasis [59].

Goblet cells and Paneth cells, as specialized intestinal epithelial cells, participate in copper handling in contexts related to immune defense and metal homeostasis, though mechanistic insights remain limited. In goblet cells, regional specification is influenced by BMP signaling, with antimicrobial peptide genes such as RegIIIγ being upregulated at the villus tip, potentially contributing to copper-dependent immune defense [59]. Copper overload induces oxidative stress in goblet cells, downregulating copper chaperone genes such as *COX17* and *ATOX1*, thereby impairing intracellular copper buffering capacity [60].

## 3. Multidimensional Regulation of Gut Health by Copper Homeostasis

### 3.1. Core Regulation at the Molecular and Cellular Level

Copper is essential for key enzymes that regulate intestinal cell function, especially in regulating redox. In COX, copper enables mitochondrial energy metabolism by coupling the mediation of electron transfer kinetics during oxygen reduction with proton translocation to drive ATP synthesis [61,62]. Beyond energy metabolism, copper-dependent SOD1 catalyzes superoxide dismutation, thereby critically protecting intestinal epithelial cells from oxidative damage. The SOD1 activity is modulated by copper availability through interactions with chaperones, such as ATOX1 and CCS; copper deficiency impairs antioxidant defenses, while its overload disrupts SOD1 dimerization via COMMD1 (Copper Metabolism Domain Containing 1) [63,64,65]. Copper further regulates gut homeostasis through amine oxidases. The activity of diamine oxidase (*AOC1*), which catalyzes the degradation of extracellular histamine in intestinal tissues, is copper-dependent and implicated in histamine intolerance and oncogenesis [66,67,68]. On the other hand, LOXL4 (Lysyl Oxidase Homolog 4) mediates extracellular matrix (ECM) stabilization via collagen/elastin cross-linking and affects cancer progression [69,70,71]. Additionally, hephaestin is a copper-dependent ferroxidase, which enables dietary iron efflux from duodenal enterocytes. Its expression is regulated by iron status and genetic loss, causing enterocytic iron retention [72,73]. Collectively, these copper-dependent enzymes integrate intracellular processes with the extrinsic microenvironmental regulation in the gut (Table 1).

Copper homeostasis functions as the central regulator of intestinal signaling pathways that govern both iron absorption and barrier integrity. DMT1 mainly mediates iron transport [81]; however, emerging evidence suggests it may also facilitate copper uptake under certain physiological conditions. A study using Belgrade rat models (b/b vs. +/b) demonstrated that the brush border membrane vesicles of DMT1-deficient mice exhibited significantly impaired copper uptake under non-energized conditions, while the copper uptake was maintained in energized vesicles through the ATP-dependent transport mechanisms [82]. These findings suggest that DMT1 may function as a secondary copper importer during specific conditions, such as copper excess.

The physiological relationship between iron and copper metabolism further complicates the assessment of DMT1 function. Iron deficiency modulates expression of copper transporters; however, the exact role of DMT1 in copper transport remains controversial [83,84,85,86]. Everted gut sac experiments showed that iron deficiency in +/b rats significantly increased copper absorption, which was completely abolished in b/b mutants [87]. These findings provided functional evidence that DMT1 could mediate adaptive copper uptake during iron-deficient conditions. Copper regulates intestinal iron absorption by stabilizing hypoxia-inducible factor-2α (HIF-2α), while HIF-2α activates the expression of iron transporters, including DMT1 and Ferroportin 1 (FPN1), during iron deficiency. Under these conditions, copper enrichment in enterocytes upregulates iron transporter expression by potentiating the DNA-binding activity and stability of HIF-2α, thereby establishing a positive feedback loop between copper and iron metabolism [10].

In the intestinal system, copper and iron metabolism are closely intertwined, primarily mediated by copper-dependent ferroxidases such as hephaestin and ceruloplasmin. Multi-copper ferroxidases (MCFs), including ceruloplasmin (CP), hephaestin (HEPH), and Zyklopen (ZP), facilitate transcellular iron efflux by oxidizing Fe^2+^ to Fe^3+^, thereby enabling iron binding to transferrin [88]. Among these, HEPH functions predominantly in the small intestine and is essential for dietary iron absorption; CP promotes iron release from the liver and central nervous system; and ZP is involved in placental iron transport [88,89,90]. All these enzymes require copper as a cofactor, and their activity is dependent on copper incorporation [89,91].Copper deficiency profoundly disrupts iron homeostasis. Insufficient copper supply reduces the activity of MCFs such as HEPH and CP, impairing iron oxidation and consequently inhibiting iron efflux via FPN from tissues including intestinal epithelial cells and macrophages into the circulation [91,92,93]. Although intestinal and hepatic iron concentrations may be elevated under copper deficiency, iron cannot be effectively mobilized into the bloodstream, leading to systemic iron deficiency and anemia [91,92,94]. At the molecular level, copper deficiency upregulates HIF-2α, enhancing the expression of duodenal iron absorption genes such as *Dmt1* and *Dcytb*, which exacerbates tissue iron retention [93]. Furthermore, copper deficiency impairs mitochondrial function, particularly the activity of CCO, the terminal complex of the electron transport chain. CCO assembly depends on copper chaperones such as COX19 [95,96]. Insufficient mitochondrial copper supply under deficiency conditions compromises CCO activity, contributing to energy metabolism dysfunction [95,97]. The synthesis of iron–sulfur (Fe-S) clusters is a core mitochondrial function. Mitochondria generate Fe-S cluster intermediates via the iron–sulfur cluster assembly machinery, which are exported for use by the cytosolic iron–sulfur protein assembly system [97]. Copper indirectly supports Fe-S cluster stability by participating in the activation of Fe-S cluster-associated enzymes such as superoxide dismutase [98]. Copper deficiency or disrupted iron metabolism impairs Fe-S cluster biogenesis, affecting mitochondrial respiratory chain function and DNA repair, ultimately leading to reactive oxygen species accumulation and cellular damage [98,99].

In addition to its role in iron metabolism, copper homeostasis critically regulates intestinal barrier integrity through interconnected pathways. Dietary copper stabilizes the HIF-1α signaling pathway, enhancing the expression of epithelial tight junction (TJ) proteins, such as occludin, and boosting antioxidant defenses, such as Glutathione Peroxidase 1 (GPX1). This stabilization strengthens the barrier and mitigates oxidative stress; this mechanism is crucial for counteracting damage in alcoholic liver disease models, where alcohol depletes HIF-1α, TJs, and antioxidants, causing barrier disruption. Notably, copper supplementation can reverse these detrimental effects, while its deficiency can exacerbate the condition [100]. In vitro studies using Caco-2 cells confirmed the copper-mediated promotion of cell growth and reduced ROS generation, supporting antioxidant-dependent barrier protection [100]. However, excessive copper intake disrupts barrier function indirectly by depleting beneficial bacteria, including *Lactobacillus johnsonii*, within the gut microbiota. This hinders the microbial conversion of acetate to butyrate, depriving colonocytes of their primary energy source, butyrate, and thereby directly impairing barrier integrity. Such damage could be reversed through supplementation with either *L. johnsonii* or butyrate [101]. Furthermore, copper modulates intestinal lipid metabolism via AMPK (AMP-activated protein kinase) activation with concomitant suppression of SREBP1 (sterol regulatory element-binding protein 1) and PPARG (peroxisome proliferator activated receptor gamma) signaling pathways, thus inhibiting intestinal lipogenesis and lipid absorption while promoting lipid transport to reduce lipid deposition. These alterations in membrane lipid composition can impact epithelial cell structure and barrier function, as demonstrated in fish models [102].

### 3.2. Shaping the Gut Microbiota

#### 3.2.1. Direct Antimicrobial Mechanism

**Figure 2:**
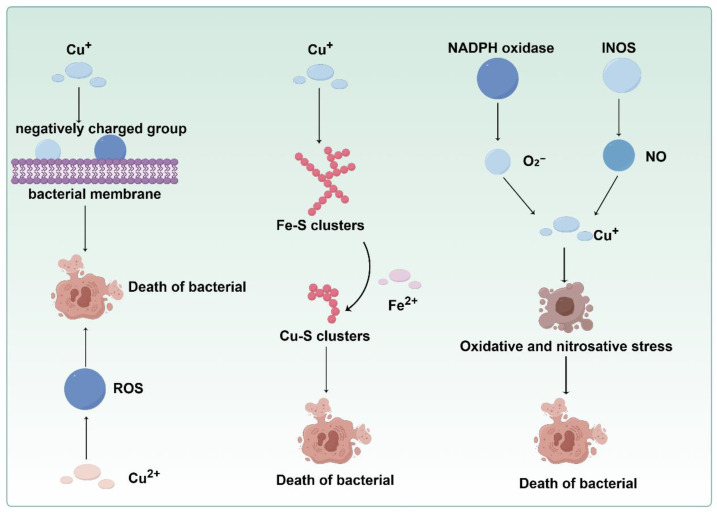
Antimicrobial mechanisms of copper. Abbreviations: ROS: Reactive Oxygen Species; INOS: inducible Nitric Oxide Synthase; NO: Nitric Oxide.

Copper exerts its broad-spectrum antimicrobial effects through multiple synergistic mechanisms. First, it directly damages bacterial cell membranes via electrostatic interactions with electronegative components (such as thiols and carboxyl groups), thus compromising membrane integrity [103]. Second, copper primarily exists as Cu^+^ within the bacterial cytoplasm and catalyzes the generation of lethal ROS and reactive nitrogen species (RNS). Host-derived superoxide (O_2_^−^) and nitric oxide (NO) undergo Cu^+^-induced redox cycling, thereby depleting bacterial antioxidants and inducing severe oxidative/nitrosative stress [104,105]. Furthermore, according to the Irving–Williams series, Cu^+^ competitively displaces essential metals (such as Fe^2+^ from [Fe-S] clusters) in metalloproteins, disrupting critical enzymatic activities and metabolic pathways [106,107,108]. This was demonstrated in *Neisseria gonorrhoeae*, where Cu^+^ could inhibit heme biosynthesis, impairing bacterial defenses [104,109] (Figure 2).

Copper ions exert dual effects on the host antimicrobial defense through their cytotoxic properties and essential functions. The high thiol affinity of Cu^+^ and ROS-generating capacity of Cu^2+^ can synergistically damage cellular constituents, such as proteins, lipids, and nucleic acids, ultimately causing lethal injury in microbial cells [110,111,112]. Moreover, copper is a crucial component of the innate immune system. Early studies suggest that copper can contribute to antimicrobial activity within phagocytes. Proinflammatory stimuli, such as IFN-γ (Interferon-gamma) and lipopolysaccharide (LPS), promote copper uptake in macrophages by upregulating CTR1 expression [113]. Concurrently, these stimuli induce ATP7A expression. This suggests the direct delivery of copper to phagocytosed bacteria. An impairment of ATP7A could reduce the bactericidal activity of macrophages against pathogens, such as *Escherichia coli* and *Salmonella Typhimurium*. Conversely, bacterial mutants deficient in copper exhibit enhanced susceptibility to ATP7A-dependent macrophage killing, further supporting the role of copper in phagocyte-mediated antimicrobial defense [114,115].

Copper plays a pivotal role at the host–pathogen interface as part of nutritional immunity, a host defense strategy that manipulates essential metal availability to inhibit microbial growth. Host cells employ distinct copper-dependent mechanisms depending on the infection niche and pathogen type. Within phagocytic cells like macrophages, copper is actively compartmentalized into phagosomes via ATP7A-mediated transport, creating a copper-rich bactericidal environment that generates toxic ROS [116,117]. Conversely, in extracellular niches such as mucosal surfaces or during adaptive immune responses, hosts sequester copper through metal-chelating proteins like calprotectin (S100A8/S100A9), S100A7, and S100A12, limiting its bioavailability to pathogens [118,119]. This dual strategy—copper intoxication in phagosomes and copper restriction in extracellular compartments—highlights the context-dependent nature of copper manipulation in host defense [120].

Pathogens have evolved sophisticated copper resistance and acquisition systems to counter host-imposed copper stress, demonstrating a co-evolutionary arms race. For detoxification, bacteria utilize copper efflux pumps such as P1B-type ATPases (e.g., CopA in *Salmonella* and *E. coli*), Cus systems for periplasmic copper export, and metallochaperones like CueP to mitigate copper toxicity [116,121]. Fungal pathogens like *Histoplasma capsulatum* and *Cryptococcus neoformans* upregulate copper exporters (e.g., CRP1) and chaperones to survive in copper-loaded phagosomes [122,123]. Simultaneously, pathogens deploy high-affinity copper acquisition mechanisms under copper-limiting conditions. *Mycobacterium tuberculosis* produces diisonitrile lipopeptides (chalkophores) that scavenge copper [124], while *Neisseria gonorrhoeae* and *Histoplasma* express copper importers (e.g., Ctr3) regulated by copper-sensing transcription factors like Mac1 to maintain essential cuproenzyme function [122,125,126]. This adaptive flexibility allows pathogens to thrive across varying host microenvironments.

The co-evolution of host copper manipulation and microbial resistance systems is evident in functional redundancies and compensatory adaptations. *Salmonella enterica* lost the ancestral CusCFBA efflux system but acquired the periplasmic chaperone CueP, which functionally substitutes for efflux under anaerobic conditions, illustrating evolutionary trade-offs [127]. Similarly, microbial siderophores, like yersiniabactin, initially evolved for iron acquisition, have been repurposed to bind copper, providing dual protection against host-imposed metal toxicity and restriction [128,129]. Host metal-sequestering proteins such as S100A7 and calprotectin exert selective pressure that drives pathogen gene diversification, as seen in the specificity of *Neisseria* TdfJ for human S100A7 [130]. This dynamic interplay underscores how microbial copper homeostasis systems are virulence factors directly shaped by host nutritional immunity [131,132].

#### 3.2.2. Impact on Microbial Composition and Its Metabolites

Dietary copper supplementation exerts dose- and source-dependent effects on gut microbiota, with strikingly distinct outcomes between physiological copper concentrations and pharmacological or excess exposure.

Animal studies have demonstrated that pharmacological or excess copper supplementation (such as 187.5–250 mg/kg diet) can significantly alter gut microbiota, as evidenced by increased ileal mucosal microbiota in poultry models. Moreover, supplementation with nano-form copper at much lower doses (1.7 mg/kg)—still representing a pharmacological intervention rather than physiological intake—could induce substantial taxonomic shifts. This was characterized by a decreased Firmicutes-to-Bacteroidetes (F/B) ratio, a pattern often associated with gut microbiota dysbiosis [133,134,135,136,137]. Similarly, in other mammalian models such as swine, high levels of dietary CuSO_4_ (175–250 mg/kg) could reduce the abundance of beneficial lactic acid-producing bacteria and decrease overall microbial diversity. Moreover, high copper levels (160 vs. 15 mg/kg) from either sulfate or hydroxy-chloride sources could induce distinct shifts in porcine gut microbiota. These changes included increased abundances of *Methanosphaera* and *Roseburia*, coupled with decreased abundances of *Fibrobacter* and *Acidaminococcus* [138].

Direct evidence regarding copper effects on the human gut microbiota remains scarce, and the translatability of animal findings to human physiology requires further investigation. In healthy children, low-dose, physiological copper exposure (e.g., 2 mg/L) enhances the abundance of beneficial probiotics such as Lactobacillus and Lactococcus in the intestinal microbiota, activates antioxidant and detoxification pathways, and contributes to the maintenance of intestinal homeostasis. However, exceeding a threshold into excess exposure (≥4 mg/L) disrupts microbial balance, reduces probiotic abundance, enriches potential pathogens, and suppresses the expression of genes involved in metabolism and detoxification, ultimately leading to intestinal barrier damage and inflammatory responses [139].

Key genes involved in copper homeostasis include *copA* and *cueO*, which function synergistically in bacterial systems to mitigate copper toxicity through efflux and oxidation pathways [140,141,142,143]. *CopA* encodes a P1B-type ATPase that actively exports excess copper from the cytoplasm to the periplasm or extracellular space. In *Escherichia coli*, CopA is regulated by the transcriptional activator CueR, which responds to elevated copper concentrations by promoting *copA* expression [143,144]. This efflux mechanism prevents cytoplasmic copper accumulation, which can lead to oxidative stress and enzyme inactivation. In pathogens like *Listeria monocytogenes*, CopA cooperates with the metallochaperone CopZ to fine-tune copper efflux, highlighting the complexity of regulatory networks [144]. Similarly, in *Deinococcus radiodurans*, the copper-responsive cluster copA-copZ-csoR ensures copper homeostasis, with CsoR acting as a repressor that dissociates from DNA upon copper binding [145]. *CueO* encodes a multicopper oxidase that oxidizes toxic cuprous ions (Cu^+^) to less reactive cupric ions (Cu^2+^) in the periplasm. This enzyme utilizes oxygen as an electron acceptor, coupling copper detoxification to oxidative processes [141,142]. Structural studies reveal that CueO contains a methionine-rich domain for copper binding and a catalytic T1 copper center, which can undergo reductive inactivation under high copper conditions [141]. While CueO is primarily associated with aerobic copper resistance, recent evidence indicates its functional role in anaerobic environments, where it complements efflux systems like CusCBA to maintain copper homeostasis [142]. Beyond these core genes, copper resistance often involves additional genetic elements. For instance, the cop operon in *Pseudomonas aeruginosa* includes *copG*, which forms a cysteine-bridged tetranuclear copper cluster to facilitate redox cycling and efflux [146]. Horizontally acquired loci like *copXL* in *Staphylococcus aureus* confer hyper-resistance via a novel efflux ATPase (CopX) and lipoprotein (CopL), enhancing survival in macrophage environments [147].

In addition to altering microbial composition, dietary copper also affects the functional metabolism of gut microbiota [148]. Physiologically relevant copper deficiency reduces the production of short-chain fatty acids (SCFAs), especially butyrate [149]. Butyrate is well known to strengthen intestinal barrier function, serving both as a primary energy source for colonocytes via β-oxidation and as a signaling molecule [150]. Conversely, excess copper levels can also induce gut microbiota dysbiosis that leads to reduced SCFA concentrations. This suggests a complex, U-shaped relationship between copper status and microbial metabolic homeostasis that warrants further investigation [150].

The effects of copper are highly dependent on dosage and chemical form, contributing to inconsistencies in the literature. Organic copper, such as copper citrate, has been shown to reduce SCFAs while potentially mitigating the spread of antibiotic resistance genes [151,152]; in contrast, inorganic copper (e.g., CuSO_4_) at 160 mg/kg improves growth performance in pigs but increases the risk of microbiota dysbiosis [138,152]. Nano-copper, even at a low dose of 1.7 mg/kg, induces significant taxonomic shifts and exhibits higher toxicity than ionic copper [153,154]. Furthermore, the role of copper in antimicrobial resistance remains debated: some studies suggest that copper exposure promotes horizontal transfer of antibiotic resistance genes via oxidative stress and mobile genetic elements [155], while others report that therapeutic levels (250 μg/g) do not significantly induce antibiotic resistance genes or mobile genetic elements [156]. The mechanistic link between copper deficiency and infection susceptibility remains unclear, and microbiota-mediated immune modulation—such as butyrate signaling pathways—requires further metabolomic validation [153]. In addition, the selective inhibition of Neisseria gonorrhoeae by copper has not been systematically compared across different species [153], highlighting a gap in cross-species mechanistic understanding.

The gut microbiota is also a fundamental instructor of the host immune system. Therefore, copper-induced dysbiosis from excess or pharmacological exposure can indirectly cause significant immune dysregulation. Microbial metabolites, such as SCFAs, modulate the differentiation and function of various immune cells, including T lymphocytes, B cells, and NK (natural killer) cells [157,158]. Evidence for microbiota-mediated immunomodulation is observed in both copper deficiency and copper excess. Copper deficiency can impair immune responses and increase infection risk through unknown microbial interactions [153]. In Wilson’s disease, a specific dysbiosis (increased Bacteroidetes abundance and decreased Firmicutes/Proteobacteria abundance) correlates with immune dysregulation [159]. Furthermore, studies in poultry models suggested that high copper levels could alter intestinal morphology and increase mucosal lymphocyte counts; these effects were likely secondary to primary alterations in microbial populations [160]. While copper clearly shapes immune responses by modulating gut microbiota, the precise mechanistic pathways, including altered metabolite production or direct microbial-host crosstalk, represent a critical frontier for future research.

### 3.3. Regulating Intestinal Immunity

**Figure 3:**
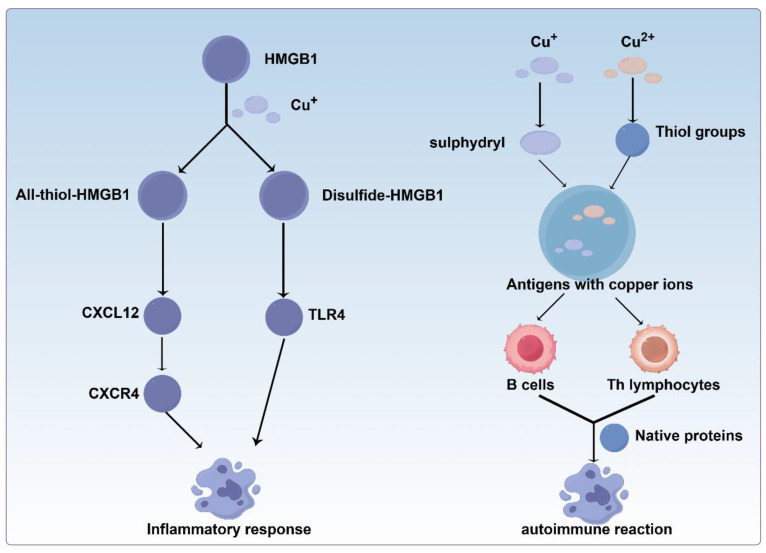
Mechanisms of copper ions affecting intestinal immunity. Abbreviations: HMGB1: High Mobility Group Box 1; CXCL12: Chemokine C-X-C Motif Ligand 12; TLR4: Toll Like Receptor 4; CXCR4: Chemokine C-X-C Motif Receptor 4.

The mechanism by which copper in the intestine affects the immune system is similar to that in other parts of the body. Copper plays a dual role in the regulation of autoimmunity. On one hand, it promotes autoimmune responses by facilitating the formation of altered self-proteins that disrupt immune tolerance. Specifically, copper ions rapidly bind to plasma proteins and biological thiols, such as cysteine and GSH, through high-affinity interactions with their sulfhydryl groups. This binding can modify native proteins, generating neo-antigens that activate B cells with help from Th lymphocytes (such as Th1 and Th2 subsets). Such activation may lead to autoimmune reactions through cross-reactivity with self-proteins [161,162,163]. On the other hand, copper may also exert immunosuppressive effects, as observed in Wilson’s disease, where copper overload paradoxically attenuates immune activation. This effect is potentially mediated by inflammation-induced copper-binding proteins, such as ceruloplasmin, which help maintain immune homeostasis by scavenging ROS and modulating immune cell activity. These mechanisms support gut barrier integrity and systemic immune defense. Additionally, copper can also directly bind to MHC (major histocompatibility complex) class II molecules and T cell receptors, possibly modulating immune responses via the idiotype-anti-idiotype network [164] (Figure 3).

High Mobility Group Box 1 (HMGB1) is a key molecular mediator linking copper metabolism to autoimmune pathogenesis. Its immunomodulatory activity depends on the redox modifications at three cysteine residues (C23, C45, and C106). Complete oxidation abolishes its function, while partial oxidation differentially activates either TLR4 (Toll Like Receptor 4)-mediated cytokine production or CXCL12-CXCR4 (C-X-C Motif Chemokine Ligand 12-C-X-C motif chemokine receptor 4)-dependent chemotaxis [165,166]. HMGB1 was initially recognized for its role in acute inflammation; however, it is now also implicated in chronic autoimmune diseases, most extensively in rheumatoid arthritis. Its elevated levels in synovial fluid and serum, along with enhanced tissue expression, contribute to inflammation via cytokine release, leukocyte recruitment, cellular activation, and autoantibody generation [167]. These effects involve multi-receptor engagement (such as TLR2/4/9) [168,169] and are amplified through HMGB1’s ability to form complexes with PAMPs (Pathogen-Associated Molecular Patterns), nucleic acids, and cytokines, thus synergistically enhancing inflammatory signaling [170].

Notably, copper homeostasis also affects intestinal immunity and barrier function. Copper supplementation can enhance intestinal structural integrity by increasing villus height, reducing crypt depth [171], and upregulating TJ proteins (such as claudin 15) and mucosal factors (such as mucin 2 and trefoil factor 3) [172]. Copper also promotes homodimerization of trefoil factor 1, facilitating mucosal repair [173]. Within immune cells, IFN-γ upregulates copper uptake in macrophages. ATP7A activation promotes phagosome-lysosome fusion, enhancing bacterial killing [114]. Adequate copper levels help in balancing the inflammatory responses by reducing pro-inflammatory cytokines, such as TNF-α ((tumor necrosis factor-α) and IL-6 (Interleukin-6) [174,175].

Both copper deficiency and excess disrupt immune homeostasis. Excess copper catalyzes hydroxyl radical formation via the Haber-Weiss reaction, damaging cellular structures and activating transcription factors, such as NF-κB (nuclear factor kappa-B) and AP-1 (Activator Protein-1), which promote expression of IL-1β, IL-8, and TNF-α [128,129]. Concurrent GSH depletion exacerbates oxidative stress [176]. In contrast, copper deficiency induces immunosuppression; this is characterized by thymic atrophy, splenomegaly, reduced neutrophil function, decreased CD4^+^ T cells, impaired IL-2 secretion, and suppressed antibody production [177,178]. Excess copper also causes shortening of the duodenal villi, lipid peroxidation, and compromised intestinal barrier integrity, which are further aggravated by hepatic GSH depletion [179]. In Monopterus albus, copper exposure induced histological damage to the intestinal epithelium, characterized by an increased number of erythrocytes and goblet cells in the lamina propria, as well as separation of the lamina propria. These structural alterations were accompanied by significant changes in the expression of genes involved in the tight junction complex, including ZO-1, Claudin-3, Claudin-12, and Claudin-15 [180]. Similarly, in rats, copper exposure compromised the structural integrity of the intestinal epithelium, resulting in a significant downregulation of both genes and proteins related to the intestinal barrier, such as Zonula occludens-1 (ZO-1), Claudin-1, and Occludin [181]. These findings indicate that copper exposure disrupts intestinal barrier function across species by targeting tight junction complexes, though the specific molecular components affected may vary.

Copper directly modulates immune cell function through metabolic reprogramming. In macrophages, mitochondrial copper ions catalyze NAD(H) redox cycling, driving glycolytic metabolic reprogramming and promoting pro-inflammatory phenotypic differentiation; targeted intervention of mitochondrial copper reverses this process and inhibits inflammatory activation [35]. Additionally, copper serves as a signaling molecule that directly binds to the intracellular pattern recognition receptor ALPK1, enhancing its sensitivity to bacterial metabolite ADP-heptose and thereby amplifying innate immune responses [182]. In antigen-presenting cells, aberrant copper concentrations interfere with HLA-I/II molecule expression, affecting T cell activation [183]. Copper also influences the immunomodulatory functions of innate lymphoid cells and B cells by altering microbial composition [180,184]. Mucin MUC2 functions as an intestinal copper chaperone, utilizing divalent copper-binding sites to prevent copper-induced oxidative cycling, protect the intestinal mucosal barrier, maintain copper bioavailability, and balance the interaction between microbiota and immune cells [185].

Pathogenic microorganisms contribute to this complex interplay by evolving copper resistance mechanisms, including efflux systems, membrane modifications, and copper chelation. These mechanisms can facilitate the co-selection of multidrug resistance [186,187]. Thus, the disruption of copper homeostasis can initiate a pathogenic cascade involving dysbiosis, chronic immune activation, and impaired mucosal immunity, thereby fostering a microenvironment conducive to oncogenic transformation in the gut.

The immunomodulatory effects of copper exhibit significant species specificity. Aquatic organisms, such as fish and turtles, suffer from more severe intestinal damage due to copper exposure, with immunosuppressive thresholds considerably lower than those observed in mammals [188,189]. In humans, patients with Wilson disease, who carry ATP7B mutations, experience copper accumulation in the intestine, leading to mitochondrial dysfunction and local inflammation. However, whether copper supplementation confers immunological benefits in the general population remains inconclusive [190]. Furthermore, although intestinal Mucin 2 can chelate copper ions to prevent oxidative damage [185], its expression levels are influenced by genetic factors, and inter-individual variability may compromise the immunoprotective effects of copper.

Copper ions generate ROS through the Fenton reaction, thereby activating the Nrf2/HO-1 antioxidant pathway. In inflammatory bowel disease models, copper-luteolin nanocomplexes have been shown to alleviate oxidative damage by modulating the Nrf2 pathway, while simultaneously suppressing NF-κB-mediated inflammatory cascades and restoring intestinal barrier function [191]. Additionally, copper influences lipid metabolism through the regulation of the PPARγ pathway. In the context of gut-liver axis disruption associated with non-alcoholic fatty liver disease, copper overload leads to inhibited β-oxidation and subsequent lipid deposition, thereby exacerbating intestinal inflammation [192].

Overall, copper exerts context-dependent immunomodulatory effects; both its deficiency and excess contribute to loss of immune tolerance and inflammatory pathogenesis. Taken together, these highlight the importance of maintaining copper homeostasis to prevent autoimmune dysregulation.

### 3.4. Systemic Metabolic Crosstalk

Copper homeostasis is centrally regulated by the liver through coordinated intestinal absorption and biliary excretion. Its expression is upregulated during copper deficiency to promote uptake [193]. Under high copper intake, ATP7A expression increases to enhance delivery to the liver [194]. Once in the portal blood, copper binds to serum proteins, such as albumin, for hepatic uptake. ATP7B facilitates copper incorporation into ceruloplasmin for systemic distribution [195,196]. Intestinal ATP7A is regulated through an organ-specific reciprocal manner: its abundance increases in enterocytes during systemic copper deficiency to boost absorption but decreases in peripheral tissues, such as the heart and spleen, to conserve copper. This pattern is reversed upon copper supplementation. Thus, the intestine is positioned as the critical gatekeeper modulating copper levels via non-autonomous regulation of ATP7A [197]. Studies in *Caenorhabditis elegans* demonstrated that in response to changes in copper levels, the subcellular localization of intestinal copper transporter CUA-1 dynamically shifted to the basolateral membrane during copper deficiency to promote efflux to tissues and relocated to lysosome-like organelles called gut granules during copper excess for sequestration and detoxification, thereby supporting the importance of intestinal copper sorting. The loss of CUA-1 disrupted copper accumulation and increased toxicity sensitivity [58]. Copper homeostasis is intricately linked to neuroendocrine signaling within the brain–gut axis, playing a critical role in both neurological and gastrointestinal physiology. The gut–brain axis mediates bidirectional communication between the central nervous system and the gastrointestinal tract via the vagus nerve and neuropeptides, such as cholecystokinin. Disruptions in copper homeostasis may interfere with this signaling pathway, thereby impairing gastrointestinal function and potentially exacerbating neuropathological processes [198]. Therefore, maintaining copper homeostasis is critical. This balance is precisely regulated through intestinal absorption, hepatic processing, biliary excretion, and peripheral utilization. Any disruption by genetic, environmental, or acquired factors can lead to systemic disorders, affecting multiple organ systems.

## 4. Dysregulation of Copper Homeostasis in Pathologies

### 4.1. Deficiency Syndromes

Pathogenic mutations in *ATP7A* gene lead to Menkes Disease (MD), an X-linked recessive disorder. This condition is characterized by impaired copper absorption, resulting in systemic copper deficiency and severe neurological and multisystem dysfunction [199]. Copper-dependent enzyme dysfunction constitutes a core mechanism underlying systemic pathology. Impaired intestinal copper absorption leads to reduced activity of multiple copper-dependent enzymes, including lysyl oxidase, tyrosinase, and cytochrome c oxidase [200]. Insufficient lysyl oxidase activity results in connective tissue abnormalities, manifesting as skin laxity, joint hyperextensibility, bladder diverticula, and defective elastin cross-linking in vascular walls [201,202]. The latter contributes to increased vascular fragility, characterized by spontaneous hemorrhage, arterial aneurysms (e.g., splenic or iliac artery aneurysms), and intracranial vascular tortuosity [203,204]. Pigmentary abnormalities arise from tyrosinase dysfunction. This enzyme catalyzes melanin synthesis, and its decreased activity leads to hypopigmentation of hair, presenting as characteristic “steely” or “kinky” hair, with microscopic evidence of twisted hair shafts (pili torti) [202,205]. Gastrointestinal dysfunction manifests as intestinal malabsorption, gastroesophageal reflux, and diaphragmatic hernia, which are associated with connective tissue abnormalities and impaired intestinal smooth muscle function [206,207]. Studies have reported more than 311 disease-causing mutations in *ATP7A*, including single-exon deletions and whole-gene rearrangements. Moreover, studies have identified a notable mutational hotspot within a 700 bp region encompassing exons 7–10, which accounts for a significant fraction of cases [208,209,210,211]. The loss of functional ATP7A protein disrupts systemic copper transport, leading to inactivation of copper-dependent enzymes. Tyrosinase, a crucial enzyme for melanin biosynthesis, exhibits markedly reduced activity in MD due to copper deficiency. Restoration of wild-type ATP7A protein could rescue tyrosinase function, confirming its copper-dependent regulation [47]. Similarly, Peptidylglycine α-Amidating Monooxygenase (PAM), required for neuropeptide amidation, has demonstrated significantly impaired activity in *ATP7A*-deficient models, contributing to the neurodevelopmental deficits observed in Menkes Disease [212]. Diminishing the activity of intestinal copper-dependent enzymes leads to the disruption of essential physiological functions, particularly nutrient absorption, antioxidant defense mechanisms, and cellular signaling pathways. Concurrently, the pathological copper accumulation in cytosolic and mitochondrial compartments disrupts redox homeostasis through Fenton reaction-mediated ROS production. This oxidative stress cascade leads to GSH depletion and mitochondrial membrane destabilization. The resulting oxidative stress further damages membrane-associated proteins, compromising ATP7A stability, thereby creating a vicious cycle that exacerbates intestinal epithelial dysfunction and systemic metabolic impairment [213].

The treatment of MD presents significant challenges, as early supplementation with copper salts, such as copper histidinate, can elevate serum copper levels; however, it demonstrates limited efficacy in alleviating neurological symptoms due to its poor penetration of the blood–brain barrier [214]. Copper histidinate is the first FDA-approved treatment for Menkes disease, improving copper absorption and utilization in affected children. In response, novel therapeutic agents are under investigation. Copper chelators, such as ES (Elesclomol) compounds, could mitigate pathological features in animal models, though their clinical applicability requires further validation [215]. Alternative strategies that focus on copper transport mechanisms, including gene therapy or small molecule modulators targeting copper-transporting proteins such as Copper Metabolism Domain Containing 1 (COMMD1) activators, represent promising future directions [216].

### 4.2. Toxicity-Driven Pathologies

Wilson disease is an autosomal recessive disorder caused by loss-of-function mutations in the *ATP7B* gene, which encodes the hepatocyte-specific copper-transporting P-type ATPase critical for systemic copper homeostasis [217]. The loss-of-function mutations in *ATP7B* (with >700 pathogenic variants reported) can disrupt copper transport activity, leading to defective biliary copper excretion and impaired ceruloplasmin maturation [218]. This functional impairment causes progressive toxic copper accumulation (not bound to ceruloplasmin) in both hepatic and extrahepatic tissues, ultimately inducing hepatic damage and systemic copper overload [219]. Wilson disease damages target organs through mechanisms involving oxidative stress, inflammatory responses, and direct cytotoxicity. Brain injury is primarily characterized by neuroinflammation and metal-induced cell death pathways [220,221]; renal damage manifests as tubular dysfunction with secondary obstruction [222]; and hematopoietic system involvement is marked by hemolysis and impaired synthetic function [223,224].

Traditional diagnostic approaches for Wilson disease primarily rely on indirect biochemical markers. Although serum ceruloplasmin measurement is widely used, it is susceptible to interference from factors such as inflammation [225,226]. Twenty-four-hour urinary copper excretion, serving as a classic indicator, exhibits decreased sensitivity in the presence of liver injury [226,227]. However, the simultaneous detection of multiple biomarkers, including Cu^2+^, pyrophosphate, and alkaline phosphatase, using a single fluorescent probe has not been achieved until recently. A novel fluorescent switch, (E)-8-((4-methylbenzylidene) amino) napthalen-1-amine (L), has been developed for the sequential, selective detection of these targets in vitro (in living cells and synovial fluid), showing alternating fluorescence responses. Given the correlation among these biomarkers in Wilson’s disease, this probe holds significant potential for future in vitro screening applications [228]. The future therapeutic directions for Wilson’s disease continue to build upon established first-line treatments, which include copper chelators (such as penicillamine and trientine) and zinc agents that inhibit intestinal copper absorption [229,230]. Beyond these conventional approaches, experimental strategies are emerging, notably involving small molecular copper chaperones, such as Elesclomol, which improve copper distribution and metabolic balance [231]. In patients with Wilson disease who present with acute liver failure, or who have progressed to decompensated cirrhosis and fail to respond to standard copper chelation therapy, liver transplantation is the indicated treatment option [232].

### 4.3. Carcinogenic Effect

**Figure 4:**
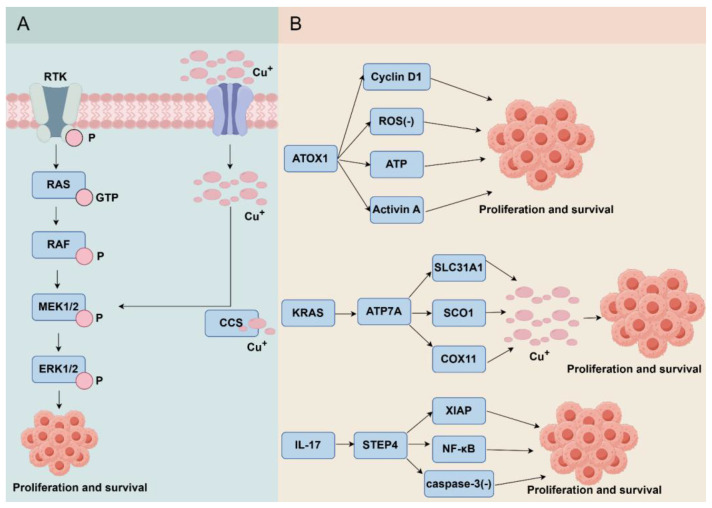
Effects of copper on cancer cell proliferation. (**A**) Copper potentiation of oncogenic signaling. (**B**) Key copper-dependent mechanisms in intestinal cancer. Abbreviations: RAS: Rat Sarcoma Viral Oncogene Homolog; RAF: Rapidly Accelerated Fibrosarcoma; MEK1/2: Mitogen-Activated Protein Kinase 1/2; ERK1/2: Extracellular Signal-Regulated Kinase 1/2; CCS: Copper Chaperone for Superoxide Dismutase; ATOX1: Antioxidant 1; ROS: Reactive Oxygen Species; ATP: Adenosine Triphosphate; KRAS: Kirsten Rat Sarcoma Viral Oncogene Homolog; ATP7A: ATPase Copper Transporting Alpha; SLC31A1: Solute Carrier Family 31 Member 1; SCO1: Synthesis of Cytochrome C Oxidase 1; COX11: Cytochrome c Oxidase Copper Chaperone COX11; IL-17: Interleukin-17; STEP4: Striatal-Enriched Protein Tyrosine Phosphatase 4; XIAP: X-linked Inhibitor of Apoptosis Protein; NF-κB: Nuclear Factor Kappa-Light-Chain-Enhancer of Activated B Cells; “(+)”: indicates positive regulation; “(-)”: negative regulation.

Dysregulation of copper homeostasis has been implicated in the pathogenesis of various malignancies, including colorectal cancer (CRC), breast cancer, hepatocellular carcinoma, and cervical cancer. Copper has emerged as a key regulator in oncogenesis and tumor progression through “cuproplasia”, which is a copper-dependent progression of cell growth mediated by both enzymatic and non-enzymatic processes [233]. Copper ions can function as allosteric regulators and potentiate oncogenic proliferation by direct binding to MEK (MAPK/ERK Kinase, Mitogen-Activated Protein Kinase-Extracellular Signal-Regulated Kinase) 1/2 kinases, which triggers the activation of the RAF (Raf kinases)-MEK-ERK signaling pathway and subsequent ERK1/2 hyperphosphorylation [233,234]. In cancer, this mechanism may position copper ionophores (such as ES) as therapeutic agents by inducing oxidative stress and degrading oncoproteins (such as p53); however, excessive copper conversely supports malignant cell survival [235]. Moreover, malignant tissues and patient sera have demonstrated elevated copper levels compared to healthy controls, with copper levels showing positive correlation with both disease progression and histological grade [236]. This copper dysregulation is further evidenced by progressively increasing serum Cu/Zn ratios across advancing CRC stages, showing strong associations with tumor aggressiveness [237]. At the molecular level, copper promotes oncogenic survival by modulating nutrient-sensing pathways via ULK (UNC-51-like kinases) 1/2 regulation [238,239]. Dysregulated copper homeostasis plays a pivotal role in CRC progression through three interconnected pathways. First, dysregulation of ATOX1 disrupts copper homeostasis, resulting in the accumulation of this copper-binding transcription factor that subsequently promotes tumorigenic processes. ATOX1 promotes cell proliferation by upregulating cyclin D1 while simultaneously suppressing ROS and enhancing ATP production [240,241]. The interaction with proinflammatory cytokines, such as activin A, further facilitates nuclear localization in CRC cells, amplifying oncogenic signaling [242]. Notably, *KRAS* (Kirsten ratsarcoma viral oncogene homolog) mutations in CRC cause copper addiction, as these mutations stabilize ATP7A copper transporters on the cell membrane, leading to a dependence on increased intracellular copper for survival [243]. Chaperones (such as SLC31A1, SCO1, COX11) channel copper into tumors, thereby further intensifying this copper reliance [244]. Inflammatory signaling engages copper metabolism via the IL-17-STEAP4 axis. Inflammatory cytokines induce STEAP4-mediated copper uptake. STEAP4 functions as a copper reductase, converting extracellular cupric ions (Cu^2+^) to cuprous ions (Cu^+^), facilitating their cellular uptake via copper transporters [245,246]. Consequently, inflammation, driven by IL-17 and other cytokines, leads to a significant accumulation of intracellular copper within tissues like the colon and lung [245,246]. This copper overload is not merely a bystander effect but plays an active pathogenic role. Elevated intracellular copper levels activate specific signaling molecules, most notably X-linked inhibitor of apoptosis protein (XIAP). Copper-dependent activation of XIAP enhances NF-κB signaling, a key pro-inflammatory and pro-survival pathway, while simultaneously suppressing caspase-3 activity, thereby inhibiting apoptosis [245]. These mechanisms promote both tumor cell survival and proliferation (Figure 4).

The CRC metastasis heavily relies on copper-dependent pathways, particularly through epithelial–mesenchymal transition (EMT), ECM remodeling, and angiogenesis. The copper-binding proteins are central to EMT initiation, where copper enables LOX to inhibit E-cadherin expression via the activation of focal adhesion kinase [247,248]. Simultaneously, copper facilitates HIF-1α interactions with hypoxia response elements, promoting the transcription of EMT regulators, such as ZEB1 (Zinc Finger E-Box Binding Homeobox 1), ZEB2, and Snail through the copper chaperone CCS [249]. ECM remodeling is also a copper-dependent process, in which LOX crosslinks collagen and elastin, thereby stiffening the matrix [250]. This mechanical change enhances FAK (Focal Adhesion Kinase) phosphorylation and fosters premetastatic niche formation, thereby correlating with increased tumor aggressiveness [251]. HIF-1α further amplifies LOX activity via the PI3K (Phosphatidyqinositol-3 kinase)/Akt (protein kinase B) pathway, establishing a feedforward loop that maintains metastatic progression [252,253]. Copper is essential for regulating angiogenesis, which supports metastatic growth. Copper activates HIF-1 to upregulate pro-angiogenic factors, such as Vascular Endothelial Growth Factor (VEGF) and ceruloplasmin, while enhancing endothelial nitric oxide synthase activity to boost vasodilatory NO production [254,255]. ATOX1 promotes angiogenesis through two mechanisms: (1) transporting copper to ATP7A for LOX activation and (2) transcriptional cofactor activity for NADPH (Nicotinamide Adenine Dinucleotide Phosphate Hydrogen) oxidase [256]. Conversely, CTR1 depletion disrupts copper uptake in endothelial cells, impairing VEGF signaling and halting neovascularization [257]. Collectively, these interrelated mechanisms position copper homeostasis as a crucial axis in the pathogenesis of CRC, presenting potential therapeutic targets.

Serum copper levels are significantly elevated in patients with colorectal cancer (CRC) compared to healthy controls, accompanied by an increased serum copper/zinc ratio, and these systemic alterations correlate positively with tumor size. Patients harboring large-volume tumors exhibit the highest serum copper concentrations alongside the lowest zinc levels, suggesting that copper–zinc imbalance may serve as a potential biomarker for tumor burden [258]. However, copper content within cancerous tissues remains a subject of debate: some studies report lower copper levels in tumor tissues compared to adjacent normal tissues, potentially reflecting rapid copper consumption by cancer cells [259]; conversely, other evidence indicates that cancer cells actively accumulate copper through upregulation of copper transporters such as CTR1 to support proliferative demands [260].

## 5. Therapeutic Targeting of Copper Homeostasis

### 5.1. Copper Chelators

Copper chelators have emerged as promising anticancer strategies by targeting copper-dependent pathways, inducing unique cell death and enhancing chemotherapy. The clinically available copper chelators, including Tetrathiomolybdate (TTM), D-penicillamine, and trientine, exert antitumor effects by depleting the bioavailable copper pools [261]. TTM acts through multiple mechanisms. It inhibits angiogenesis by downregulating NF-κB, SOD1, and HIF-1α levels [262]. Additionally, TTM could reverse *BRAF* (B-Raf Proto-Oncogene, Serine/Threonine Kinase)^V600E^-mediated chemoresistance in mutant CRC models by blocking the RAS-RAF-MEK-ERK pathway [263]. D-penicillamine and trientine exhibit similar mechanisms, impairing metastatic potential by disrupting tumor vascularization and ECM maturation [264]. The synthetic chelator TPEN (N,N,N′,N′-Tetrakis-[2-Pyridylmethyl]-Ethylenediamine) has demonstrated selective CRC cytotoxicity through the ROS-mediated cell death pathways [265,266]. In the future, mechanistic studies should be expanded to elucidate processes, such as the interaction between cuproptosis and EMT. In parallel, selective delivery systems require optimization, and individualized clinical trials must advance to ultimately enable precision therapy.

Clinical translation is underway, with PET imaging using [64Cu] CuCl2 enabling noninvasive monitoring of copper metabolism and TTM efficacy in triple-negative breast cancer (TNBC) [267]. Early human studies indicate TTM reduces intestinal copper absorption, limiting exposure to the liver and brain [268]. Ongoing trials explore TTM in combination regimens, such as with lenvatinib for hepatocellular carcinoma, where it suppresses VEGF expression and microvessel density [269]. TTM’s toxicity profile is manageable but requires careful monitoring. It reduces serum ceruloplasmin activity (a biomarker of copper status) without significant hepatotoxicity in animal models [270,271]. However, prolonged copper depletion may impair physiological processes, as copper is essential for superoxide dismutase and cytochrome c oxidase activity [272,273]. In Wilson’s disease patients, TTM effectively lowers copper burden but can cause reversible neurological effects if overdosed [268] (Table 2).

### 5.2. Copper Ionophores

Copper ionophores promote intracellular copper accumulation, thereby inducing oxidative stress and cell death through cuproptosis [284]. Copper ionophores leverage the unique biological features of the tumor microenvironment to achieve selective antitumor effects. Tumoral tissues exhibit significantly elevated levels of copper as compared to normal tissues, thereby providing a foundational basis for copper ionophore activity [285]. Furthermore, these agents exploit the overexpression of specific receptors on cancer cells, such as the asialoglycoprotein receptor, to enhance drug enrichment preferentially within malignant cells [284]. Additionally, cancer cells rely on mitochondrial respiration for energy production. Copper ionophores induce mitochondrial copper overload, subsequently disrupting cellular energy metabolism and amplifying their cytotoxic effects [286]. Disulfiram (DSF), clioquinol, and bis (thiosemicarbazone) analogs demonstrate this approach. Originally developed to treat alcoholism, DSF exhibits potent anticancer activity by inhibiting oncogenic signaling pathways, including NF-κB and MAPK signaling pathways [287,288]. DSF-Cu complexes can induce autophagy and suppress cancer stemness, highlighting their multifaceted role in CRC therapy [289,290]. Clioquinol, a derivative of chloroquine, can clear XIAP, a protein that regulates apoptosis, thereby sensitizing CRC cells to cell death [277]. ES mediates its anticancer activity through copper-dependent cytotoxicity. ES facilitates intracellular copper accumulation by forming ES-Cu complexes. This triggers dual cytotoxic mechanisms: oxidative stress-induced apoptosis and cuproptosis through direct interference with TCA cycle components and ferredoxin 1 activity [278]. Bis(thiosemicarbazones) exert their antitumor effects through multiple mechanisms. They induce apoptosis in cancer cells via both the mitochondrial pathway, characterized by reduced mitochondrial membrane potential and upregulated caspase-3/7 activity, and the death receptor pathway, evidenced by enhanced caspase-8 activation [291]. Additionally, these compounds induce cell cycle arrest by modulating the expression of cyclin-dependent kinases and cyclins, thereby trapping tumor cells in specific phases of the cell cycle [292,293]. Bis(thiosemicarbazones) also suppress tumor migration and invasion by downregulating matrix metalloproteinase expression and inhibiting epithelial–mesenchymal transition [292]. Notably, these compounds exhibit potent radical-trapping antioxidant activity, inhibiting lipid peroxidation and conferring resistance to ferroptosis [292]. These multifaceted mechanisms contribute to the antitumor activity of bis(thiosemicarbazones) against various cancer types, including bladder cancer [292], breast cancer [294], lung cancer [295], and prostate cancer [296].

DSF-Cu and clioquinol-copper complexes represent promising repurposed therapeutic strategies in oncology, with extensive preclinical investigation but limited clinical success to date. The active metabolite, copper diethyldithiocarbamate (CuET or Cu(DDC)_2_), is central to its cytotoxicity, but its hydrophobicity and rapid metabolism of oral DSF impede bioavailability and tumor accumulation [297,298,299]. Despite robust preclinical data, clinical trials face challenges. A phase Ib study in metastatic castration-resistant prostate cancer (mCRPC) revealed that oral DSF rapidly metabolizes to inactive diethyldithiocarbamate-methyl ester (Me-DDC), failing to achieve efficacy despite copper supplementation [300]. Similarly, a phase II trial in recurrent temozolomide-resistant glioblastoma showed DSF-Cu was well-tolerated but offered minimal clinical benefit, underscoring limitations in unselected populations [301]. To overcome metabolic instability, nanoformulations like stabilized Cu(DDC)_2_ nanoparticles (SMILE method) and liposomal Cu(DDC)_2_ are advancing, demonstrating high drug loading, stability, and efficacy in drug-resistant models [302,303]. Clioquinol (CQ), an antifungal agent, forms a copper complex (Cu(CQ)_2_) with anticancer potential. However, its poor aqueous solubility and rapid metabolism hinder therapeutic utility. Liposomal Cu(CQ)_2_ formulations enable intravenous delivery but showed limited efficacy in glioblastoma and ovarian cancer models, either alone or combined with DSF [304]. Thus, while mechanistic insights support CQ’s potential, effective delivery systems remain critical for translation.

### 5.3. Nanoparticle Therapeutics

Nanoparticle therapeutics play a pivotal role in copper-targeted cancer treatment, primarily by inducing cuproptosis and enhancing radiotherapy sensitivity, as well as their synergistic application in photothermal/photodynamic therapy (PTT/PDT). Copper-based nanoparticles (such as copper sulfides or copper oxides) and copper-ion-loaded nanocarriers (such as liposomes and metal–organic frameworks) enable efficient delivery of copper into tumor cells, thus overcoming the limitations of free copper ions, such as poor targeting and short half-life. For instance, acid-degradable copper hydride nanoparticles can selectively release copper ions within the tumor microenvironment to trigger cuproptosis [305]. Similarly, the combination of transferrin-templated copper nanoclusters (Tf-CuNCs) with doxorubicin could significantly suppress tumor growth and prolong survival in animal models [306]. Furthermore, copper-based nanoparticles, such as ^64^Cu-labeled CuS, act as radiosensitizers by generating free radicals under localized irradiation, thereby synergistically increasing DNA damage [307]. Additionally, due to their localized surface plasmon resonance properties, copper-based nanomaterials exhibit strong near-infrared absorption and excellent photothermal conversion efficiency. In the tumor microenvironment, endogenous hydrogen peroxide (H_2_O_2_) reacts with Cu^2+^ ions to produce oxygen (O_2_), alleviating tumor hypoxia, which is a critical barrier to oxygen-dependent therapies like PDT [308,309,310]. This catalytic decomposition of H_2_O_2_ not only mitigates hypoxia but also supplies O_2_ for subsequent ROS generation, enhancing therapeutic efficacy [308,310]. Simultaneously, elevated GSH levels in tumors reduce Cu^2+^ to Cu^+^, depleting GSH and weakening the cellular antioxidant defense system [308,311,312]. The depletion of GSH is crucial, as it minimizes the scavenging of ROS and amplifies oxidative stress [311,313]. The resulting Cu^+^ ions then catalyze a Fenton-like reaction with H_2_O_2_, generating highly toxic hydroxyl radicals (•OH) that induce oxidative damage to cellular components, including lipids, proteins, and DNA [309,314,315]. The Fenton-like reaction represents an improvement and extension of the classical Fenton reaction, employing alternative transition metal ions (such as copper or manganese), supported solid catalysts, or external energy inputs (e.g., light or electricity) to replace or assist ferrous ions in catalyzing hydrogen peroxide to generate highly oxidative hydroxyl radicals. The combination of copper-based nanoparticles with PTT/PDT offers precise spatial control and multi-modal therapeutic capacity, enabling targeted tumor ablation, and exhibits enhanced therapeutic efficacy [316].

Copper nanoparticles show promise in cancer therapy due to their unique properties: enhanced tumor targeting via the EPR effect and surface functionalization; improved systemic stability with tumor-specific controlled release; and multimodal therapeutic effects that activate multiple cell death pathways and enable synergistic therapies, overcoming drug resistance and targeting cancer stem cells. But their clinical application hinges on three key factors: biocompatibility, systemic toxicity, and clearance. Biocompatibility is largely determined by surface coatings—such as Polyethylene glycol or biomimetic membranes—which improve stability and tumor targeting while reducing immune recognition and off-target effects [317,318,319]. Toxicity stems mainly from copper ion release, which can induce cancer cell death via mechanisms like cuproptosis but may also cause organ damage if not properly controlled [320,321,322,323]. Clearance pathways depend on particle size and surface chemistry: small, hydrophilic particles are rapidly cleared by the kidneys, while larger ones are processed by the liver and spleen [324,325,326]. Optimizing surface engineering and dosing is essential to balance therapeutic efficacy with safety.

## 6. Conclusions and Future Prospects

Copper homeostasis critically orchestrates intestinal health through the regulation of enzymatic catalysis, microbiota composition, barrier integrity, and immune function. In the context of malignancy, the dysregulation of this delicate balance drives tumor progression. As outlined in this review, this occurs through the activity of copper-dependent enzymes, which promote oxidative stress and metastasis, while the disruption of copper transport by key mediators such as CTR1, ATP7A, ATP7B, and the chaperone ATOX1 underpins chemoresistance and tumor survival. Furthermore, the recent elucidation of cuproptosis, a distinct cell death mechanism triggered by mitochondrial copper accumulation and the aggregation of lipoylated TCA cycle proteins, has revealed a novel therapeutic vulnerability. Exploiting this mechanism, emerging strategies including copper ionophores and engineered copper nanoparticles offer a means to selectively induce cancer cell death. These approaches represent a paradigm shift in targeting the dual role of copper in both proliferation, termed cuproplasia, and cytotoxicity.

Understanding how copper homeostasis shapes the gut microbiome represents a critical frontier for future investigation. This interaction presents a promising axis for investigation, as shifts in microbial populations could serve as a systemic link influencing both intestinal health and the efficacy of antitumor immunity. To translate these findings into clinical practice, developing multimodal biomarkers will be essential. Such biomarkers should not only facilitate patient stratification based on tumor copper status but also enable dynamic monitoring of therapeutic responses to copper-targeted interventions. Key translational steps include optimizing delivery systems for copper chelators and ionophores to enhance tumor specificity, rigorously profiling the safety and toxicity of nanoparticle-based therapies, and exploring rational combination approaches such as integrating copper modulation with established chemotherapies or immune checkpoint inhibitors to overcome drug resistance and potentiate antitumor immune responses. Ultimately, targeting copper homeostasis represents a promising and multifaceted axis poised to yield both fundamental mechanistic insights and transformative translational applications in the fight against cancer.

## Figures and Tables

**Table 1:** Function and cell-specific expression of Cu-dependent enzymes in the intestine.

Enzyme	Function	References
Cytochrome c oxidase	Electron transfer and oxygen reduction, proton pumping and energy conversion, and structural features and metal centers	[74]
Cu/Zn-dependent superoxide dismutase 1	Antioxidant defense, immune regulation, inflammatory response, and gene transcription regulation	[75]
Cu-dependent amino-oxidase 1 (diamine oxidase)	Diamine metabolism and maintenance of intestinal barrier function	[76,77]
Peptidylglycine α-amidating monooxygenase	Catalysis of the amidation reaction	[78]
Lysyl oxidase homologue 4	Deamination of lysine and hydroxylysine residues of collagens and elastin	[79]
Heparin	Ferroxidase: facilitates iron efflux from the cell	[80]

**Table 2:** Mechanisms underlying various therapeutic strategies targeting copper homeostasis.

Category	Name	Mechanisms	Result	Clinical Status	References
Copper chelators	Tetrathiomolybdate	Sequesters copper ions; downregulates NF-κB/SOD1/HIF-1α; targets RAS-RAF-MEK-ERK pathway	Inhibits angiogenesis; reverses chemoresistance (BRAF^V600E^ mutants)	clinical trial	[261,262,263]
	D-penicillamine/Trientine	Disrupts tumor vascularization and collagen cross-linking	Reduces metastasis	clinical trial	[264,274]
	TPEN	Induces oxidative stress	Selectively kills cancer cells	preclinical	[275]
Copper ionophores	Disulfiram	DSF/Cu complexes inhibit NF-κB/MAPK pathways; induces autophagy; suppresses cancer stemness	Inhibits tumor growth	preclinical	[276]
	Clioquinol	Clears XIAP (apoptosis regulator)	Sensitizes cancer cells to death	preclinical	[277]
	Elesclomol	ES-Cu complexes transport Cu^2+^ into cells; induces oxidative stress/TCA cycle disruption	Triggers cuproptosis	preclinical	[278]
Nanoparticle Therapeutics	Copper-loaded nanoparticles	Induces cuproptosis, enhances traditional therapies, synergizes with PTT/PDT, and regulates the immune microenvironment	Induces cuproptosis; suppresses immune evasion, distant metastasis, and recurrence	preclinical	[279,280,281,282,283]

Abbreviations: NF-κB: nuclear factor kappa-B; BRAF^V600E^: a specific mutation in the BRAF gene, which encodes a protein involved in cell growth and division. TPEN: N,N,N′,N′-Tetrakis-[2-Pyridylmethyl]-Ethylenediamine; SOD1: Cu/Zn-Superoxide Dismutase 1; HIF-1α: Hypoxia-Inducible Factor 1α; DSF: Disulfiram; XIAP: X-linked Inhibitor of Apoptosis Protein; ES: Elesclomol; TCA: Tricarboxylic Acid; PTT: photothermal therapy; PDT: photodynamic therapy.

## Data Availability

No new data were created or analyzed in this study.

## Abbreviations

AOC1	Amine Oxidase 1;
ATOX1	Antioxidant 1 Copper Chaperone;
ATP7A	ATPase copper transporting alpha;
CCS	Copper Chaperone for Superoxide Dismutase;
COMMD1	Copper Metabolism Domain Containing 1;
COX11	Cytochrome c Oxidase Copper Chaperone 11;
COX17	Cytochrome c Oxidase Copper Chaperone 17;
CP	ceruloplasmin;
CRC	Colorectal Cancer;
CTR1	Copper Transport protein 1;
CTR2	Copper Transport protein 2;
CXCL12	Chemokine C-X-C Motif Ligand 12;
CXCR4	Chemokine C-X-C Motif Receptor 4;
DMT1	Divalent Metal Transporter 1;
FPN1	Ferroportin 1;
GPX1	Glutathione Peroxidase 1;
DSF	Disulfiram;
ECM	Extracellular Matrix;
EMT	epithelial–mesenchymal transition;
ER	Endoplasmic Reticulum;
ES	Elesclomol;
ETC-derived	Electron Transport Chain-derived);
GSH	Glutathione;
HEPH	hephaestin;
HIF-1α	Hypoxia-Inducible Factor 1α;
HMGB1	High Mobility Group Box 1;
INOS	Inducible Nitric Oxide Synthase;
LOXL4	Lysyl Oxidase Homolog 4;
MD	Menkes Disease;
MTs	Metallothioneins;
MCFs	Multi-copper ferroxidases;
NF-κB	Nuclear Factor Kappa-Light-Chain-Enhancer of Activated B Cells;
PAM	Peptidylglycine α-Amidating Monooxygenase;
PAMPs	Pathogen-Associated Molecular Patterns;
PD-L1	Programmed cell death ligand 1;
ROS	Reactive Oxygen Species;
SCFAs	Short-Chain Fatty Acids;
SCO1	Synthesis of Cytochrome C Oxidase 1;
SCO2	Synthesis of Cytochrome C Oxidase 2;
SLC11A2	Solute Carrier Family 11 Member 2;
SLC31A1	Solute Carrier Family 31 Member 1;
SLC31A2	Solute Carrier Family 31 Member 2;
SOD1	Cu/Zn-Superoxide Dismutase 1;
STEAP	Six-Transmembrane Epithelial Antigen of the Prostate;
STEAP4	Six-Transmembrane Epithelial Antigen of the Prostate 4;
TCA	Tricarboxylic Acid;
TGN	Trans-Golgi Network;
TLR4	Toll-Like Receptor 4;
TPEN	N,N,N′,N′-tetrakis-[2-pyridylmethyl]-ethylenediamine;
TTM	Tetrathiomolybdate;
VEGF	Vascular Endothelial Growth Factor;
XIAP	X-linked Inhibitor of Apoptosis Protein;
5-FU	5-Fluorouracil,
PTT	photothermal therapy,
PDT	photodynamic therapy.
